# Ghrelin's effects on food motivation in rats are not limited to palatable foods

**DOI:** 10.1111/jne.12665

**Published:** 2019-01-02

**Authors:** Tina Bake, Christian E. Edvardsson, Cameron J. Cummings, Suzanne L. Dickson

**Affiliations:** ^1^ Department of Physiology/Endocrine Institute of Neuroscience and Physiology, The Sahlgrenska Academy at the University of Gothenburg Gothenburg Sweden

**Keywords:** fasting, food devaluation, ghrelin, goal‐directed behaviour, hunger, operant conditioning

## Abstract

The “hunger” hormone, ghrelin, is powerfully orexigenic. Even in the absence of hunger, ghrelin delivery to rats increases consumption of chow, as well as palatable foods, and increases motivated behaviour for palatable food rewards. Inspired by the finding that ghrelin increases the selection of chow in rats offered a choice diet (lard, sucrose or chow) and even in rats bingeing on a high‐fat diet, we aimed to explore whether the effects of ghrelin on motivation extend to regular chow. Rats were conditioned to lever press for either chow or sucrose pellets in a progressive ratio (PR) operant conditioning task. The effect of acute i.c.v. delivery of ghrelin on both chow and sucrose self‐administration was determined and compared with overnight fasting (ie, when endogenous ghrelin levels are elevated). We found that ghrelin similarly increased motivated behaviour for chow and sucrose pellets. The effect of fasting on motivated behaviour for both food pellets was comparable in magnitude to that induced by ghrelin, albeit with an earlier ceiling effect during the PR session. Devaluation experiments (in which rats are offered either food reinforcer in excess prior to PR testing) did not support the hypothesis that sucrose pellets would be more difficult to devalue (as a result of their higher incentive value) than chow pellets. When exchanging the respective pellets during a PR session, chow‐conditioned rats were more motivated for sucrose pellets compared to chow pellets; however, sucrose‐conditioned rats were similarly motivated for chow pellets compared to sucrose pellets. Thus, using sucrose as a reward may increase the motivation even for less palatable foods. We conclude that the impact of ghrelin on food‐motivated behaviour in fed rats is not limited to palatable foods but extends to regular chow, and also that the magnitude of the effect is considerable compared to that of an overnight fast.

## 1 INTRODUCTION

Ghrelin, discovered almost two decades ago,^1^ is an orexigenic hormone secreted by the stomach during the preprandial period, when hungry.^2^ The growth hormone secretagogue receptor 1a (GHS‐R1a) is responsible for mediating the effects of ghrelin, and it is expressed in many different brain areas.^3^ Ghrelin acts on homeostatic pathways in the hypothalamus^4^, ^5^ and brainstem,^6^, ^7^ which are areas likely to be important with respect to any effects on food intake,^8^, ^9^ weight gain and adiposity.^10^ It also acts on reward pathways, notably the ventral tegmental area to nucleus accumbens dopamine pathway,^11^, ^12^ to promote the consumption of palatable foods.^13^, ^14^ Ghrelin impacts on a wide range of feeding‐linked behaviours: it increases food anticipatory behaviour^15^ even in fed rats anticipating a chocolate treat,^16^ is required for food reward from chocolate,^17^ and increases food‐motivated behaviour for sucrose pellets^13^, ^18^ and for a high‐fat diet.^19^

In addition to promoting the consumption of both chow^8^ and palatable food,^17^ ghrelin has also been shown to alter food choice. Considering the involvement of ghrelin in food reward and food motivation for palatable food, it is interesting that, when a food choice consisting of chow, lard and sucrose pellets is offered, acute delivery of ghrelin mainly induces a preference change towards chow.^20^ Moreover, in a scheduled‐feeding paradigm, when animals are bingeing on a high‐fat diet, acute delivery of ghrelin can change the food preference during the binge from a high‐fat diet to chow.^21^ Such work inspired the present study, which investigates whether ghrelin is also able to increase the motivation for healthier (less palatable) foods such as chow, even in satiated rats.

The present study aimed to explore the impact of acute ghrelin delivery on progressive ratio operant responding for chow pellets in rats. We hypothesised that ghrelin delivery would increase the self‐administration of chow pellets in a similar manner to self‐administration of sucrose pellets. We also compared the effect of ghrelin delivery on food‐motivated behaviour with that induced by an overnight fasting because ghrelin is a circulating hunger hormone and its endogenous levels are elevated during fasting.^22^, ^23^

## 2 MATERIALS AND METHODS

### 2.1 Animals

Forty‐eight male Sprague‐Dawley rats (Charles River, Sulzfeld, Germany) at 7 weeks of age at arrival were allowed to acclimatise in group‐housing for 1 week prior to the experimental procedures. The rats were kept under standardised nonbarrier conditions under a 12:12 hour light/dark cycle at approximately 21°C and 50% relative humidity. Rats had ad lib. access to standard maintenance chow diet (#2016; Harlan Labs, Indianapolis, IN, USA; 22% protein, 66% carbohydrate, 12% fat by energy, 3.0 kcal g^‐1^) and water unless otherwise stated. All animal experimental procedures were approved by the local animal ethics committee at the University of Gothenburg, Sweden (permit number 45‐2014).

### 2.2 Intracerebroventricular surgery

Rats were implanted with an i.c.v. guide cannula into the lateral ventricle (−0.9 mm posterior to bregma, ±1.6 mm lateral to the midline and −2.5 mm ventral of the skull surface) under anaesthesia, induced by an i.p. injection of a Ketaminol (75 mg kg^‐1^; Intervet, Boxmeer, Netherlands) and Rompun (10 mg kg^‐1^; Bayer, Leverkusen, Germany). Rats were positioned in a stereotaxic frame (Model 942; David Kopf Instruments, Tujunga, CA, USA) and the skull bone was exposed. Bregma was located and used as the origin for coordinates. Holes for guide cannulae and anchoring screws were drilled. A 26‐gauge cannula (#C32G‐SPC; Bilaney, Sevenoaks, UK) was positioned according to the coordinates and fixed in place with anchoring screws (#MCS1x2; Agnthos, Lidingö, Sweden) and dental cement (#7508, #7509; Agnthos). A dummy cannula (#C313DC; Bilaney) was inserted into the guide cannula to prevent obstruction. After surgery, the rats received an analgesic (Rimadyl; Orion Pharma Animal Health, Sollentuna, Sweden) and were housed individually. After recovery of at least 3 days, cannulae placement and projection length of the injector (2.0 or 2.5 mm) was confirmed in conscious rats with an injection of 2 μL of angiotensin II (10 ng μL^‐1^; #1158; Tocris, Bristol, UK). Cannulae placement was considered correct when a dipsogenic response occurred within 5 minutes of injection and at least 5 mL of water was consumed within 30 minutes. Rats that did not qualify the criteria were excluded for further experimental procedures.

### 2.3 Progressive ratio operant conditioning

Rats were divided into two groups in accordance with body weight for progressive ratio (PR) operant conditioning and assigned to either the chow (body weight of 319.4 ± 3.1 g; n = 24) or the sucrose (body weight of 319.3 ± 3.1 g; n = 23) lever‐pressing group. For 6 days per week, rats were then conditioned to press a lever for either 45‐mg chow pellets (#1811156; TestDiet, Richmond, IN, USA; 24% protein, 66% carbohydrate, 10% fat by energy, 3.3 kcal g^‐1^) or 45‐mg sucrose pellets (#1811251; TestDiet; 100% carbohydrate by energy, 3.4 kcal g^‐1^) using 16 rat operant conditioning chambers (Med‐Associates Inc., St Albans, VT, USA). Each chamber had a metal grid floor, a house light, two retractable levers with cue lights above them, and a pellet dispenser that can deliver 45‐mg pellets. Only one of the levers was active during conditioning and testing; pressing the inactive had no programmed consequence, although the number of inactive lever presses was recorded. The paradigm for PR operant conditioning has been described in detail previously.^24^ Briefly, the conditioning sessions occurred over 2 weeks under approximately 50% food restriction (with access to 15 g of chow diet per day at the beginning of the dark phase) with a schedule of 30 minutes of fixed ratio (FR) sessions twice a day (FR1, FR3 and FR5) and 120 minutes of PR sessions once a day. This was followed by 1 week of PR sessions without food restriction once a day until the responses were considered stable. Chow and water were withheld during the conditioning and testing sessions.

In the PR sessions, the number of presses required to receive the next reward (pellet) was progressively increased to determine the amount of work the rat is willing to put into obtaining the reward. The response requirement increased according to: response ratio = [5*e* ^injection number × 0.2^] − 5 and proceeded through the series: 1, 2, 4, 9, 12, 15, 20, 25, 32, 40, 50, 62, 77, 95, 118, 145, 178, 219, 268, 328. During PR sessions, the operant chambers were programmed to shut down automatically if a rat had failed to earn a reward within 60 minutes (defined as breakpoint), although all rats remained in the operant chambers for the entire session length of 120 minutes regardless of the session finishing earlier. During all PR sessions, we measured the active/inactive lever presses, the number of food pellets earned, the response ratio, and the locomotor activity at each 10‐minute interval time point.

### 2.4 Experimental procedures

#### 2.4.1 Experiment 1: Effect of ghrelin and food restriction on motivation for chow or sucrose

Once the rats had successfully learned to lever press for chow pellets or sucrose pellets, the effect of ghrelin on food motivation was tested. Acyl ghrelin (1 or 2 μg; #1463; Tocris) or vehicle (artificial cerebrospinal fluid; #3525; Tocris) was injected i.c.v. at a volume of 2 μL in ad lib. fed rats. The ghrelin doses had previously been shown to induce a feeding response in rats,^25^ as well as to increase motivation for sucrose self‐administration in an identical PR paradigm.^18^ The injection of ghrelin has previously been shown to have similar effects to fasting in the context of food choice.^20^ To compare the effect of ghrelin injection with fasting on chow or sucrose self‐administration, vehicle was also injected in overnight fasted rats. All injections were made 20 minutes prior to starting a PR session in a counterbalanced manner with at least 48 hours between injections, so that each rat received each of the four conditions in a randomised order (fed‐vehicle, fed‐1 μg ghrelin, fed‐2 μg ghrelin and fasted‐vehicle). Chow diet intake was measured for 1 hour after the PR session end and 24 hours post‐injection because the orexigenic effect of ghrelin typically lasts for approximately 4 hours. All injections were performed in the early or mid light phase. Measurements relating to motivation were analysed in 10‐minute sample bins with cumulative data over the 120 minutes of the PR session.

#### 2.4.2 Experiment 2: Effect of devaluation on motivation for chow or sucrose

These studies were undertaken to explore possible differences in the motivational value of sucrose vs chow, with the general expectation that sucrose likely has a higher motivational value and will be more difficult to devalue than chow. Accordingly, rats received 15 g of their respective pellets in their home cages overnight before the devaluation PR session. Another PR session without additional pellets was performed to compare with baseline conditions. Chow diet intake was measured 24 hours after the start of the PR session.

#### 2.4.3 Experiment 3: Effect of pellet exchange on chow or sucrose self‐administration

Because both chow pellets and sucrose pellets were devalued with prior overnight access in Experiment 2, we aimed to investigate the effect of exchanging pellets during one PR session; for example, rats conditioned with chow pellets had to lever press for sucrose pellets, and rats conditioned with sucrose pellets had to lever press for chow pellets. Another PR session with the regular pellets was performed to allow comparison with baseline conditions. Chow diet intake was measured 24 hours after the start of the PR session.

### 2.5 Statistical analysis

Statistical analysis was performed using spss, version 25 (IBM Corp., Armonk, NY, USA). Data in all experiments were first analysed for equal variances. Data in Experiment 1 were then analysed by repeated measures (RM)‐ANOVA with period of time as the within‐subject factor and the treatment as the between‐subject factor. The four different treatments were fed‐vehicle, fed‐1 μg ghrelin, fed‐2 μg ghrelin and fasted‐vehicle. If a general effect of the treatment as well as an interaction between time and treatment was revealed during RM‐ANOVA, a further one‐way ANOVA was conducted at each time period, followed by Tukey's post‐hoc tests to reveal effects of ghrelin injections or fasting within the chow or sucrose‐conditioned groups. Data independent of period of time were either analysed by one‐way ANOVA followed by Tukey's post‐hoc tests or by an independent samples *t* test to compare vehicle injections between chow‐ and sucrose‐conditioned groups. Data in Experiments 2 and 3 were analysed by independent samples *t* tests, separately for chow‐ and sucrose‐conditioned rats. Outliers were identified by Grubb's test and excluded separately from each experimental part. Only statistical significant outcomes (*P* < 0.05) are reported. Data are presented as the mean ± SEM.

## 3 RESULTS

### 3.1 Experiment 1: Effect of ghrelin and food restriction on motivation for sucrose or chow

**Figure 1:**
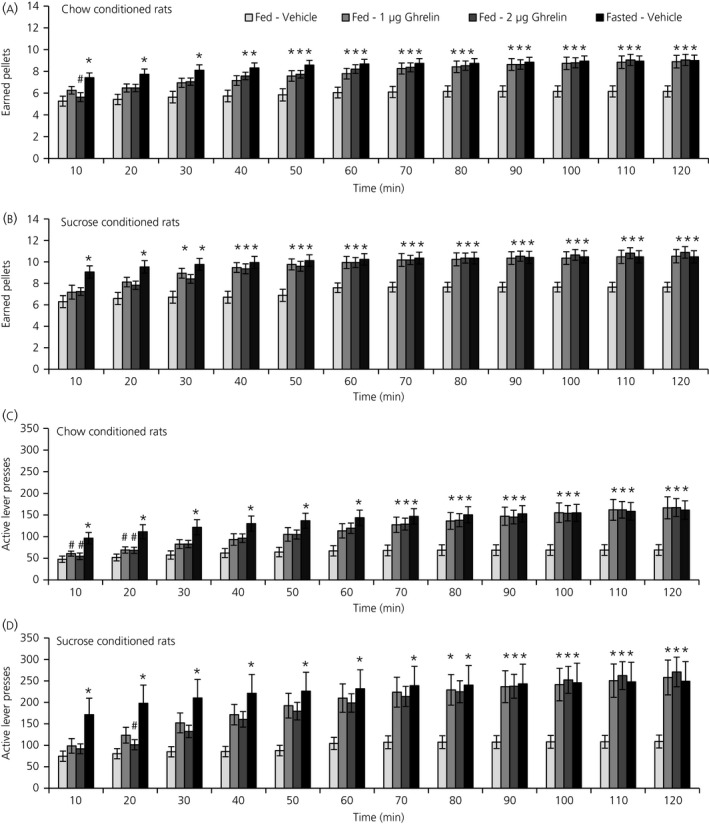
Ghrelin and fasting increase the motivation for both chow pellets and sucrose pellets in 120‐min progressive ratio operant responding sessions. The number of earned pellets is significantly increased by ghrelin and fasting in rats (A) conditioned with chow and (B) conditioned with sucrose. The number of active lever presses is also significantly increased by ghrelin and fasting in rats (C) conditioned with chow and (D) conditioned with sucrose. Data are shown as the mean ± SEM. *P <* *0.05 vs vehicle; ^#^ *P *<* *0.005 vs fasting. For clarity, *P *<* *0.01 and *P *<* *0.001 are not differentiated from *P *<* *0.05. Chow‐conditioned rats, n = 19; sucrose‐conditioned rats, n = 18

Rats conditioned with chow pellets or sucrose pellets under a PR paradigm were acutely injected with ghrelin into the lateral ventricle. RM‐ANOVA revealed a general effect of treatment (*P* = 0.001, chow; *P* < 0.001, sucrose), as well as an interaction between time and treatment (*P* = 0.005, chow; *P* = 0.012, sucrose), on the number of pellets earned (Figure 1A,B). Further one‐way ANOVA testing on each time period revealed that ghrelin significantly increased the number of pellets earned during the PR session, irrespective of whether the food reinforcer was chow (Figure 1A) or sucrose (Figure 1B). For chow self‐administration, the increase in the number of earned pellets started from 40 minutes onwards with the higher ghrelin dose (ghrelin 2 μg, *P* = 0.026) and from 50 minutes onwards with the lower ghrelin dose (ghrelin 1 μg, *P* = 0.048). For sucrose self‐administration, the increase for the number of earned pellets started from 30 minutes onwards with the lower ghrelin dose (*P* = 0.012) and from 40 minutes onwards with the higher ghrelin dose (*P* = 0.003). An overnight fast also increased the number of earned pellets during chow and sucrose self‐administration. However, in contrast to ghrelin delivery, fasting increased the number of earned pellets already during the first 10 minutes sampling bin (chow pellets, *P* = 0.003: sucrose pellets, *P* = 0.004). At session end (120 minutes), there was a 1.4‐fold increase in the number of earned chow pellets with the lower ghrelin dose (*P* = 0.003), and a 1.5‐fold increase with both the higher ghrelin dose (*P* = 0.001) or fasting (*P* = 0.002). Earned sucrose pellets were 1.4‐fold increased for both ghrelin doses and fasting compared to vehicle (ghrelin 1 μg, *P* = 0.002; ghrelin 2 μg, *P* = 0.001; fasting, *P* = 0.003).

**Figure 2:**
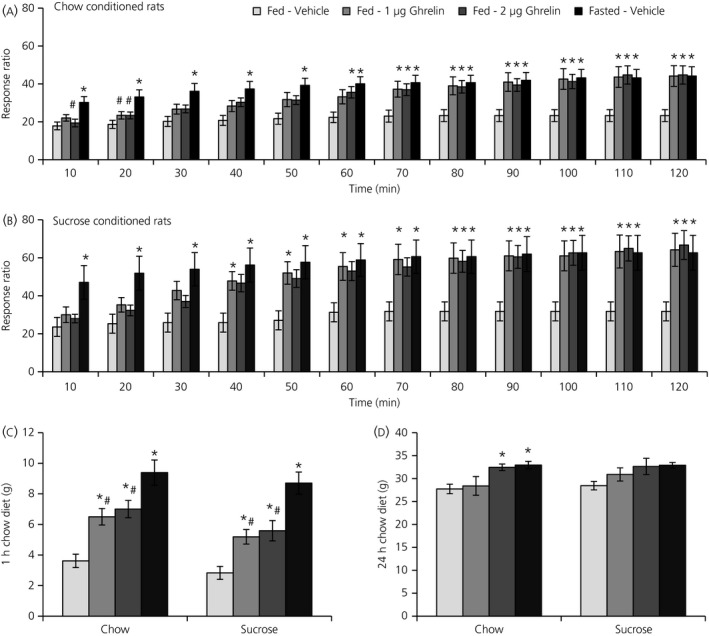
Ghrelin and fasting increase the motivation for both chow pellets and sucrose pellets in 120‐min progressive ratio operant responding sessions. The response ratio (ie, the response requirement needed to advance to the next level) is significantly increased by ghrelin and fasting in rats (A) conditioned with chow and (B) conditioned with sucrose. Ghrelin and fasting also increase the intake of chow diet (C) for 1 h after PR session in both the chow‐ and sucrose‐conditioned rats. D, 24 h after injection, chow diet intake is only increased with the higher ghrelin dose in chow‐conditioned rats. Data are shown as the mean ± SEM. *P <* *0.05 vs vehicle; ^#^ *P *<* *0.005 vs fasting. For clarity, *P *<* *0.01 and *P *<* *0.001 are not differentiated from *P *<* *0.05. Chow‐conditioned rats, n = 19; sucrose‐conditioned rats, n = 18

In both sucrose‐ and chow‐conditioned rats, RM‐ANOVA also revealed a general effect of treatment on number of active lever presses (*P* = 0.004, chow; *P* = 0.015, sucrose) and the response ratio (*P* = 0.003, chow; *P* = 0.007, sucrose), as well as an interaction between time and treatment on the number of active lever presses (*P* = 0.024, sucrose) and the response ratio (*P* = 0.017, chow; *P* = 0.044, sucrose). The interaction between time and treatment on the number of active lever presses only showed a trend in chow‐conditioned rats (*P* = 0.067). Further one‐way ANOVA testing on each time period revealed that ghrelin delivery and fasting significantly increased the number of active lever presses (Figure 1C,D) and the response ratio during the PR sessions (Figure 2A,B). The results of the post‐hoc tests following one‐way ANOVA for number of active lever presses and the response ratio showed a pattern similar to that for the number of pellets earned. Inactive lever presses and locomotor activity during the PR session did not show a general effect of treatment or an interaction of time and treatment and were therefore unaffected by ghrelin injection or fasting in both groups of rats (data not shown).

In addition, both ghrelin doses also increased chow diet intake in both chow‐conditioned rats (ghrelin 1 μg, *P* = 0.007; ghrelin 2 μg, *P* = 0.001) (Figure 2C) and sucrose‐conditioned rats (ghrelin 1 μg, *P* = 0.029; ghrelin 2 μg, *P* = 0.007), measured for 1 hour after the PR sessions. Fasting increased chow diet intake the most in both chow‐conditioned rats (*P* < 0.001 vs vehicle; *P* = 0.007 vs ghrelin 1 μg; *P* = 0.035 vs ghrelin 2 μg) and sucrose‐conditioned rats (*P* < 0.001 vs vehicle; *P* < 0.001 vs ghrelin 1 μg; *P* = 0.002 vs ghrelin 2 μg). At 24 hours after ghrelin injection, chow diet intake was still increased for chow‐conditioned rats with the higher ghrelin dose (*P* = 0.048) (Figure 2D) and with fasting (*P* = 0.024).

To compare chow self‐administration with sucrose self‐administration, the vehicle responses for all previous measurements and time points were compared. However, none of the data showed significant differences between chow and sucrose self‐administration.

### 3.2 Experiment 2: Effect of devaluation on motivation for chow or sucrose

**Figure 3:**
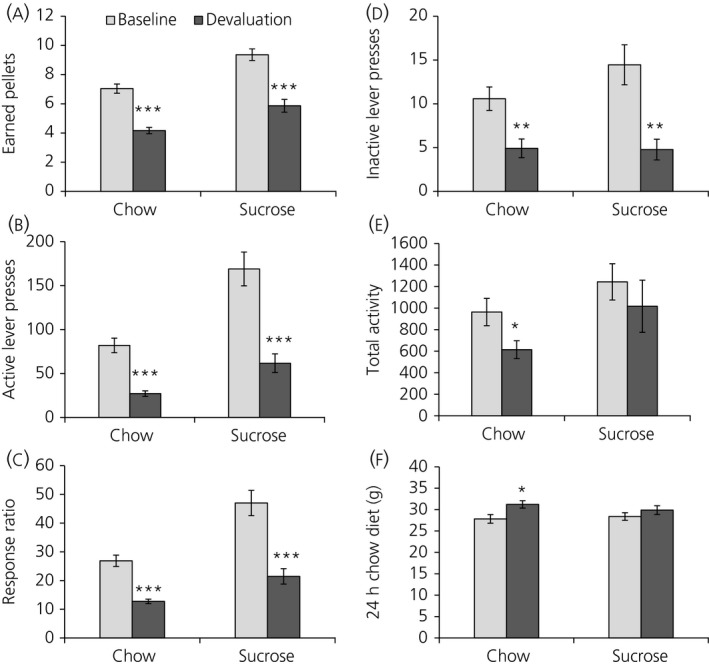
Devaluation in chow‐ and sucrose‐conditioned rats leads to a decrease in motivation parameters for both chow and sucrose pellets. A, Number of earned pellets; B, number of active lever presses; C, response ratio; D, inactive lever presses; E, total activity; F, 24‐h chow diet intake. Data are shown as the mean ± SEM. *P <* *0.05, **P <* *0.01, ***P <* *0.001. Chow‐conditioned rats, n = 24; sucrose‐conditioned rats, n = 22

Devaluation of sucrose pellets or chow pellets was carried out by giving ad lib. access to the respective pellets overnight prior to the devaluation PR session, where both groups of rats ate a similar amount of pellets (chow pellets 12.6 ± 0.6 g; sucrose pellets 13.8 ± 0.3 g; not significant). Devaluation PR compared to baseline PR led to a decrease in motivation parameters during both sucrose and chow self‐administration; there was a significant decrease in the number of earned pellets (sucrose rats, *P* < 0.001; chow rats, *P* < 0.001) (Figure 3A), the number of active lever presses (sucrose rats, *P* < 0.001; chow rats, *P* < 0.001) (Figure 3B) and the response ratio (sucrose rats, *P* < 0.001; chow rats, *P* < 0.001) (Figure 3C) in both sucrose‐ and chow‐pressing rats. Devaluation also led to a decrease in inactive lever presses (sucrose rats, *P* = 0.001; chow rats, *P* = 0.002) (Figure 3D) in both groups of rats, as well as to a decrease in locomotor activity during the PR session in chow‐pressing rats (*P* = 0.026) (Figure 3E). Chow diet intake, measured 24 hours after PR session start, was increased in rats pressing for chow (*P* = 0.014) (Figure 3F) but not for sucrose.

### 3.3 Experiment 3: Effect of pellet exchange on chow or sucrose self‐administration

**Figure 4:**
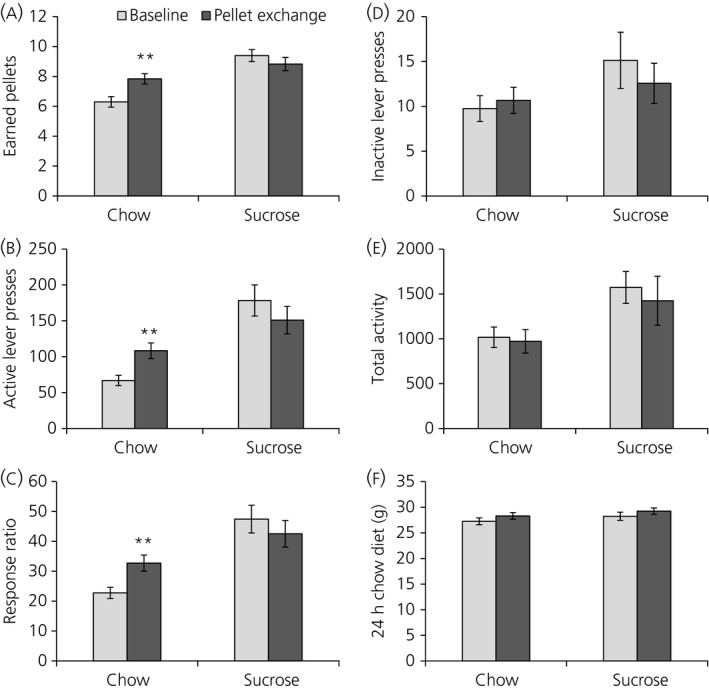
Exchanging the pellets during a progressive ratio (PR) session leads to an increase in motivation in rats conditioned with chow but pressing for sucrose. Rats conditioned with sucrose but pressing for chow do not show a different motivation. A, Number of earned pellets; B, number of active lever presses; C, response ratio; D, inactive lever presses; E, total activity; F, 24 h chow diet intake. Data are shown as the mean ± SEM. *P <* *0.05, **P <* *0.01, ***P <* *0.001. Chow‐conditioned rats, n = 24; sucrose‐conditioned rats, n = 23

To further investigate the value of the pellets, we exchanged the pellets during one PR session. Exchanging the pellets during a PR session led to an increase in motivation parameters in rats conditioned with chow pellets but pressing for sucrose pellets; there was a significant increase in the number of earned pellets (*P* = 0.003) (Figure 4A), number of active lever presses (*P* = 0.003) (Figure 4B) and the response ratio (*P* = 0.004) (Figure 4C). Rats conditioned with sucrose pellets but pressing for chow pellets did not show a change in number of earned pellets, number of active lever presses or the response ratio. Inactive lever presses (Figure 4D), locomotor activity (Figure 4E) or 24‐hour chow diet intake (Figure 4F) were not affected by the pellet exchange.

## 4 DISCUSSION

The present study was designed to investigate whether (i) acute intra‐brain delivery of ghrelin would be able to increase the motivation for bland foods such as chow pellets by using a PR operant responding paradigm and whether (ii) the effect would be similar to the effects of ghrelin on the motivation for palatable foods such as sucrose pellets. Previously, we showed that ghrelin can increase the motivation for sucrose pellets^18^ and that the target site of this effect is the ventral tegmental area.^13^ In the present study, using an identical PR paradigm, we show that ghrelin delivery into the lateral ventricle increases the motivation for chow pellets, as reflected by an increase in the number of chow pellets earned, the number of active lever presses and the response ratio of these (which indicates the amount of work/response required to earn the next reward at each time point). Moreover, the effect of ghrelin on motivation for chow pellets was very similar to its effects on motivation for sucrose pellets, in terms of the number of pellets earned, the number of active lever presses and the response ratio. Thus, our data demonstrate that the effects of ghrelin on food motivation are not limited to palatable foods but extend to include chow.

Given that endogenous ghrelin levels are elevated during fasting,^22^, ^23^ we also explored the effect of an overnight fast on food motivation. What is striking is the similarity between fasting (a very powerful stimulus for food‐motivated behaviour) and ghrelin on the level of food motivation (in terms of the number of active lever presses and pellets earned). What differs between fasting and ghrelin injection is the time course of the effect. It is perhaps not surprising that hungry rats start working for food pellets as soon as they enter the operant chamber and rapidly reach their break point (ie, the response ratio at which no further pellets are obtained), whereas the effects of ghrelin in fed rats take a longer time to emerge (approximately 30‐40 minutes), likely reflecting the time course to reach brain areas driving this behaviour. We conclude that lateral ventricle delivery of ghrelin powerfully drives food‐motivated behaviour to a level similar to that induced by an overnight fast.

A number of important controls were included in the present study, which further validate the approach used. First, we show that basal levels of motivated behaviour, in vehicle‐treated rats, were similar regardless of whether the reinforcer was sucrose or chow and remained steady throughout the experiment. We also found that ghrelin delivery increased the subsequent free feeding of chow diet in rats conditioned with either chow or sucrose as expected for an orexigenic hormone. Thus, no matter whether lever‐pressing for chow or for sucrose, this did not alter the subsequent chow intake when the rats were returned to their home cage. Finally, we addressed potential concerns that any differences in lever‐pressing in the two food conditions could reflect differences in the reward value of sucrose vs chow. We hypothesised that the palatable sucrose pellets would be more valuable than the bland chow pellets because all rats had ad lib. access to chow diet throughout all experiments and that therefore only sucrose pellets could be devalued. Accordingly, the rats were given ad lib. access to their respective pellets overnight prior to the devaluation PR session. However, both chow‐conditioned and sucrose‐conditioned rats were less motivated to self‐administer their respective pellets, as reflected by a decreased number of pellets earned, a decreased number of active lever presses and a decreased response ratio. This suggests that chow pellets and sucrose pellets have a similar incentive value to the rats, and also that lever‐pressing for either food pellet had not become habitual.

Regarding their energy density, chow pellets and sucrose pellets are very comparable (3.3 and 3.4 kcal g^‐1^, respectively); however, their macronutrient composition is very different because sucrose only contains energy from carbohydrate. The chow pellets are also very comparable to the chow diet that was offered ad lib. to all rats, both with respect to energy density and macronutrient composition. One could assume that rats do not care about eating one or the other. However, they vary hugely in size and this might explain why rats prefer the smaller “bite‐sized” chow pellets (eg, whole apple vs apple slices in a human context) such that the chow pellets could also be devalued.

Because both sucrose and chow were devalued with prior overnight access in Experiment 2, we aimed to investigate the effect of exchanging pellets during one PR session; for example, rats conditioned with sucrose pellets had to self‐administer chow pellets, and rats conditioned with chow pellets had to self‐administer sucrose pellets. As expected, rats conditioned with chow pellets were more motivated to self‐administer sucrose pellets. For rats conditioned with sucrose pellets, we expected that they would be less motivated to self‐administer chow pellets. However, the sucrose‐conditioned rats were similarly motivated when the pellets were exchanged for chow. This provides an additional confirmation that chow pellets and sucrose pellets have a similar value to the rats. Another explanation is that rats conditioned with a palatable food such as sucrose pellets will self‐administer any other food even if it is less palatable or less valuable.

We conclude that the central ghrelin signalling system targets pathways that are important for food motivation, irrespective of its caloric content or palatability. Ghrelin appears to have very powerful effects on food motivation, comparable to those induced by an overnight fast. These data contribute to the growing literature indicating that the effects of ghrelin on food‐linked behaviours are important not only for the intake of palatable/reward treats, but also for more bland foods.

## CONFLICT OF INTERESTS

The authors declare that they have no conflicts of interest.

## AUTHOR CONTRIBUTIONS

TB and SLD designed the research study. TB, CEE and CJC performed the research. TB, CEE and CJC analysed the data. TB and SLD wrote the manuscript.
